# Pathological investigation, molecular characterization and first-time isolation of the predominant serotypes of fowl adenovirus (FAdV-D and E) from commercial poultry in Bangladesh

**DOI:** 10.3389/fmicb.2024.1490255

**Published:** 2024-11-28

**Authors:** Mohammad Sadekuzzaman, Md. Sojon Miah, Rokshana Parvin, Md. Enamul Haque, Tanbin Rubaiya Islam, Sanzila Hossain Sigma, Md. Golbar Hossain, Sajedul Hayat, Muhammad Tofazzal Hossain, Md. Alimul Islam

**Affiliations:** ^1^Central Disease Investigation Laboratory, Department of Livestock Services, Dhaka, Bangladesh; ^2^Department of Microbiology and Hygiene, Bangladesh Agricultural University, Mymensingh, Bangladesh; ^3^Department of Pathology, Bangladesh Agricultural University, Mymensingh, Bangladesh; ^4^Department of Veterinary and Animal Sciences, University of Rajshahi, Rajshahi, Bangladesh

**Keywords:** fowl adenoviruses (FAdV), histopathology, PCR, phylogenetic analysis, serotypes-8b and 11

## Abstract

**Background:**

Fowl adenovirus (FAdV) is a globally distributed virus that inflicts significant economic losses on the poultry industry. The study aimed at pathological investigation, molecular characterization, isolation, and pathogenicity determination of FAdV from commercial poultry.

**Methods:**

A total of 86 liver samples were collected from 80 commercial chicken farms. All samples were examined for gross pathology; only liver samples were used for histopathology and virus detection by PCR. PCR-positive FAdV samples were used for isolation of virus in 10-day-old seronegative embryonated chicken eggs (ECEs) via chorioallantoic membrane (CAM). PCR confirmed virus isolates were sequenced for serotyping and phylogenetic analysis. Pathogenicity of the isolated FAdVs was assessed by oral, i.m., and i.p. routes of infection.

**Results:**

The study observed gross lesions including hydropericardium and enlarged, friable pale livers with ecchymotic hemorrhages. Histopathological analysis revealed hepatocytic necrosis with basophilic intranuclear inclusion bodies in liver tissues, and tubular necrosis, focal hemorrhages, and mononuclear cell infiltration in kidney tissues. Out of 86 liver samples analyzed, 40 were positive for FAdVs by PCR, and 22 were positive for virus isolation. One serotype was 11 and other 11 were 8b of FAdV and genetically close to Bangladesh, India, and Turkey isolates, according to hexon gene phylogenetic analysis. The pathogenicity test indicated that serotype 11 was more virulent than the serotype 8b.

**Conclusion:**

The study concluded that serotypes 11 and 8b of FAdVs are circulating simultaneously among commercial broiler and layer chickens, serotype 8b was found predominant one.

## 1 Introduction

There are around 319.68 million poultry populations in Bangladesh and there is an annual growth rate of 15% (DLS, 2023). The poultry sector is thriving in livestock agriculture but is facing a serious threat of various viral diseases. Among the diseases, inclusion body hepatitis (IBH) caused by fowl adenoviruses (FAdV) has emerged newly as a significant threat to commercial poultry (broilers and layers) in Bangladesh (Schachner et al., 2018). IBH is a viral disease of poultry caused by several genotypes of FAdV (A-E) (Zahid et al., 2024). FAdV is a member of the family *Adenoviridae* and has 12 distinct serotypes. These serotypes are differentiated by their non-enveloped, icosahedral nucleocapsids that consist of double-stranded DNA. Additionally, they are classified into six distinct genera of viruses *Atadenovirus, Aviadenovirus, Ichtadenovirus, Mastadenovirus, Siadenovirus*, and *Testadenovirus* (Athukorala et al., 2022**)**. Sero-types of the viruses were determined by cross-neutralization tests are FAdV-1, FAdV-2, FAdV-3, FAdV-4, FAdV-5, FAdV-6, FAdV-7, FAdV-8a, FAdV-8b, FAdV-9, FAdV-10, and FAdV-11. Serotypes 1 and 5 are composed of Fowl Adenovirus A (FAdV-A) and B (FAdV-B), respectively (Wajid et al., 2018). The serotypes of FAdV-C are 4 and 10. Serotypes 6, 7, 8a, and 8b belong to FAdV-E, and serotypes 2, 3, 9, and 11 are in FAdV-D viruses and are responsible for the deadly illness known as IBH (Marek et al., 2016). Many countries have reported IBH outbreaks, with serotypes 2, 4, 8a, 8b, and 11 being shown to be the primary causes (Chitradevi et al., 2021).

IBH has become a major health issue for the chicken industry worldwide, following its first identification in the United States in 1963. The virus has inflicted substantial economic losses in several countries, including India, Pakistan, Korea, the United States, Canada, Hungary, China, Japan, and Bangladesh, since the beginning of the outbreak (Wajid et al., 2018). The virulence of recently discovered strains, specifically FAdV 8b and 11, has been examined with different results (Sabarudin et al., 2021). Multiple studies have shown that infecting chickens of different age groups, ranging from day 1 to 3 weeks old, with FAdV serotypes 8b and 11 led to decreased clinical signs and fatality rates. Poultry species, including chickens, ducks, turkeys, geese, and pheasants, are vulnerable to FAdV infection (Abd El-Ghany, 2021). IBH is characterized by hepatocyte necrosis, the presence of intra-nuclear inclusion bodies, and a high mortality rate. The death rate reaches its highest point on the day 4^th^ or 5^th^ following infection (Sharma et al., 2018). The mortality rate of chickens affected by IBH might vary, with rates below 10% being common but the maximum rate is 100% (Gulhane et al., 2016). FAdV can be transmitted horizontally or vertically. Horizontal transmission happens through the dissemination of feces, contaminated substances, water, and the environment. Vertical transmission, on the other hand, includes infected breeder chickens spreading the virus to their offspring through eggs, namely the FAdV-4 and FAdV-8 strains (Li et al., 2017). The major mode of transmission of IBH is likely to be through asymptomatic infected hens, particularly to birds with compromised immune systems. The primary causative species of IBH in chickens are FAdV-D and FAdV-E, with serotypes 7, 8a, 8b, and 11 being the most common (Sabarudin et al., 2021).

The initial occurrence of FAdV infection in broiler parents was recorded in Bangladesh in 2002 (Biswas et al., 2002). It is thought that the disease has entered Bangladesh and is currently spreading across the poultry industry, mostly affecting broiler and layer birds. A recent study conducted in Bangladesh has discovered the presence of many serotypes (5, 8b, 11), which have been determined to be responsible for inflicting death and significant economic losses (Islam et al., 2023). The FAdV infections have a significant economic impact on the poultry industry, causing reduced productivity and increased mortality rates. The present study aims to pathological investigation, characterization, and isolation of the predominant serotypes of FAdVs from broiler and layer chickens in Bangladesh. This study provides crucial information about their prevalence and genetic diversity. Such insights will help in developing effective control and prevention strategies in future. This research is very important for providing information to the poultry sectors to protect poultry health, ensure food security, and maintain the economic stability in the poultry sector of Bangladesh.

## 2 Materials and methods

### 2.1 Study areas

**Figure 1:**
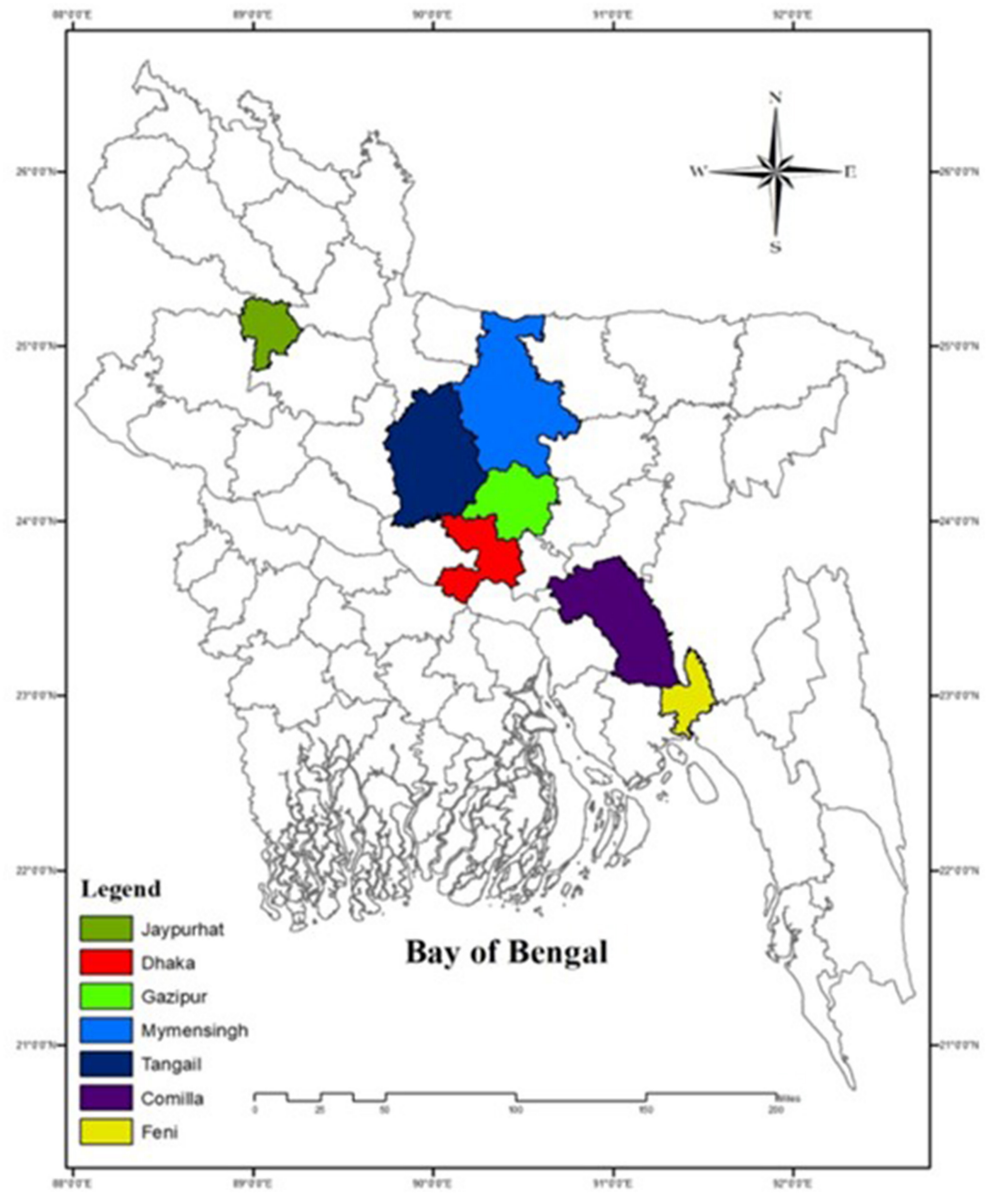
Sampling districts of Bangladesh marked by different color (ArcGIS 10.8.2. California USA).

The FAdVs suspected field samples were collected from seven districts of Bangladesh during the outbreak year 2023 to 2024, particularly from the districts of Feni (22.9409°N, 91.4067°E), Tangail (24.3917°N, 89.9948°E), Dhaka (23.7539°N, 90.3372°E), Joypurhat (25.0968°N, 89.0227°E), Gazipur (24.0958°N, 90.4125°E), Mymensingh (24.7539°N, 90.4073°E), and Cumilla (23.4576°N, 91.1809°E) (Figure 1).

### 2.2 Collection and processing of samples

In this study, a total of 86 liver samples were collected from 80 commercial poultry farms (50 from broilers and 30 from layers) of seven different outbreak districts of Bangladesh (Table 1). The samples were collected based on the clinical history, clinical signs, and postmortem examination. The flock suspected with FAdV infection showed several clinical signs, including depression, varying degrees of dullness and drowsiness, reduced body weight gain, reduced feed intake, respiratory distress, ruffled feathers, reluctant to move, lack of uniformity, diarrhea, and sudden death. Liver samples were used for histopathological study, while liver, kidney, gizzard, and spleen were examined for gross pathology through postmortem investigation. The frozen FAdV-suspected liver samples were thawed and grounded using sterile mortar and pestle with sterile 1X PBS to create a 20% (w/v) solution. The resulting suspension was clarified by centrifugation at 5,000 rpm for 20 min at 4°C. The supernatant was then collected and passed through a 0.22 μm Millipore filter. Following filtration, the filtrate was added with antibiotics (10 mg/mL streptomycin and 10,000 IU/mL penicillin) and 25 μg/mL of amphotericin-B and incubated at room temperature for 45 min. To ensure the sterility of the inoculum, the prepared inoculum was then overlaid onto a blood agar medium to check any contamination.

**Table 1:** Collection of samples from FAdVs suspected chickens of different outbreak districts of Bangladesh.

**Name of districts**	**No. of farms**		**No. of samples collected from dead and sick chickens**		**No. of liver samples collected**	
	**Broiler**	**Layer**	**Broiler**	**Layer**	**Broiler**	**Layer**
Feni	8	6	8	6	8	6
Tangail	5	3	7	5	7	5
Dhaka	7	4	13	12	10	5
Joypurhat	5	5	8	7	7	4
Gazipur	8	3	12	9	9	7
Mymensingh	10	5	6	8	6	4
Cumilla	7	4	6	7	5	3
**Total**	**50**	**30**	**60**	**54**	**52**	**34**

### 2.3 Histopathology of liver samples of FAdV suspected chicken

Liver samples were collected from the sick and freshly dead chickens infected with suspected IBH virus (IBHV). The samples were preserved with 10% neutral buffered formalin. The tissues were then dehydrated, cleaned, and embedded in paraffin before being microtomed into 4 μm thick slices (Oliver-Ferrando et al., 2017). Hematoxylin and eosin stains were used to stain this sectioned tissue, which was then seen under a light compound microscope (Olympus CX23 binocular microscope, Japan).

### 2.4 Molecular detection of FAdV

Viral DNA was extracted from 86 liver tissue samples of suspected birds using the QIAGEN mini-DNA extraction kit (QIAGEN, Germany) as per the manufacturer's instructions. The hexon gene located in the loop one region of FAdV was used for PCR amplification. Primers for this study were based on the modified method of Radwan et al. (2019). DNA samples were dissolved in 60 μL of nuclease-free water and used for PCR. The hexon gene fragment was amplified using specific primers: forward primer Hex-L1-s (5′-ATGGGAGSACCTAYTTCGACAT-3′) and reverse primer Hex-L1-as (5′-AAATTGTCCCKRAANCCGATGTA-3′), producing a 590 bp size DNA fragment. The PCR reaction was carried out in a 50 μL volume containing 5 μL of DNA template (10–30 ng/μl), 25 μL of KAPA2G Fast Ready-mix (Sigma-Aldrich, USA), 2.5 μL (1 picomol/μl) of each primer, and 15 μL of DEPC-H_2_O. The mixture was placed in a thermal cycler (T1000, Bio-Rad, USA) with the following conditions: pre-denaturation at 95°C for 10 min, 40 cycles of denaturation at 95°C for 30 s, annealing at 60°C for 45 s, extension at 72°C for 45 s, and final extension at 72°C for 10 min. The PCR products were allowed to run in a 1.5% agarose gel stained with ethidium bromide and visualized under a UV transilluminator (Bio-Rad, USA).

### 2.5 Isolation of FAdV using seronegative embryonated chicken eggs

Virus isolation was conducted using PCR-positive samples in 10-day-old seronegative embryonated chicken eggs (ECEs). Each liver tissue was allowed to homogenate and made (20%) and the 0.2 mL/embryo was inoculated into 10 ECEs via the chorioallantoic membrane (CAM) route, while 20 ECEs inoculated with phosphate buffer saline (PBS) as negative controls. The eggs were incubated at 37°C for 5 days and candled twice daily. Embryo deaths within the first 24 h were deemed non-specific and discarded. Embryos that died after 48 h and those surviving until day 5 were necropsied. CAM and allantoic fluid were harvested for PCR confirmation; however, liver and kidney tissues were collected for histopathological examination.

### 2.6 Sequencing and phylogenetic analysis

Twelve representative isolates were randomly selected for sequencing and phylogenetic analysis. Approximately 40 μL of each PCR product, containing the amplified hexon gene, was sent to Malaysia for First Base sequencing using the BigDye^®^ Terminator v3.1 Cycle Sequencing Kit (Thermo Fisher Scientific, USA). Both forward and reverse hexon primers were used for sequencing. The sequences were aligned using MEGA11 (Tamura et al., 2021). Standard FAdV hexon gene sequences from GenBank were compared with previous isolates of Bangladesh, India, and Nepal along with one out-group (Newcastle disease virus). A phylogenetic tree was constructed using the neighbor-joining method and validated with 1,000 bootstrap replicates (Horiike, 2016).

### 2.7 Pathogenicity of isolated FAdVs

#### 2.7.1 Selection of isolates of FAdVs for pathogenicity study

Of the 12 isolates, the serotypes 11 (PP897935 Alim IBH 1012) and 8b (PP897933 Alim IBH 1010) were chosen for pathogenicity assessment based on observation of gross pathology, rate of morbidity and mortality, clinical signs, and molecular identification by PCR.

#### 2.7.2 Determination of titration of virus

The virus titer was determined by injecting serial 10-fold dilutions of liver homogenate into 10-day-old seronegative ECEs via the CAM route. The study determined the median embryo infective dose (EID_50_) of the FAdVs isolate using the Reed and Muench (1938) method.

#### 2.7.3 Experimental design for pathogenicity test

A total of 105 broiler chicks (Cobb-500, day-old age), consisting of both males and females, were collected from the National Hatchery, Gazipur, Bangladesh, to study viral pathogenicity. The seronegative status of the chickens against was confirmed using an ELISA test to detect maternal antibodies against fowl adenovirus (BioChek, USA). The chickens were reared in stainless-steel cages in the small animal laboratory house, Department of Microbiology and Hygiene, Bangladesh Agricultural University, Mymensingh-2202. At 5 days of age, the chicks were randomly divided into seven groups, each group including the control group consisting of 15 birds. The test groups were inoculated with two recent isolates of FAdVs. The birds of each test group were infected with serotype 11 isolate (PP897935 Alim IBH 1012) and serotype 8b isolate (PP897933 Alim IBH 1010) at a dose of 10^4.5^ EID_50_/0.2 mL via i.p., i.m., and oral routes. The chickens in the control group were administered with equal volume of PBS via the i.m. route. All the chickens were monitored twice daily for any signs of illness of IBH until the 14^th^ day, during which clinical signs and mortality were recorded. The scoring of clinical signs was determined using the method described by Wei et al. (2023), while the day-wise mortality pattern was calculated following the approach outlined by Chavan et al. (2024). The scoring scale was as follows: 0 indicated no signs of disease; (1) indicated precursory clinical signs like depression and disorganized feathers; (2) indicated clear clinical signs, such as yellowish or greenish drooping, curled fetal position, and anorexia; and (3) indicated deaths. The clinical signs score curve and day-wise mortality curve were prepared using Microsoft Excel (Microsoft Office, version 2021). The chicken found moribund within or after 14 days of infection was euthanized and postmortem examination and collected their liver samples for molecular analysis by PCR.

## 3 Results

### 3.1 Clinical and postmortem investigation

**Figure 2:**
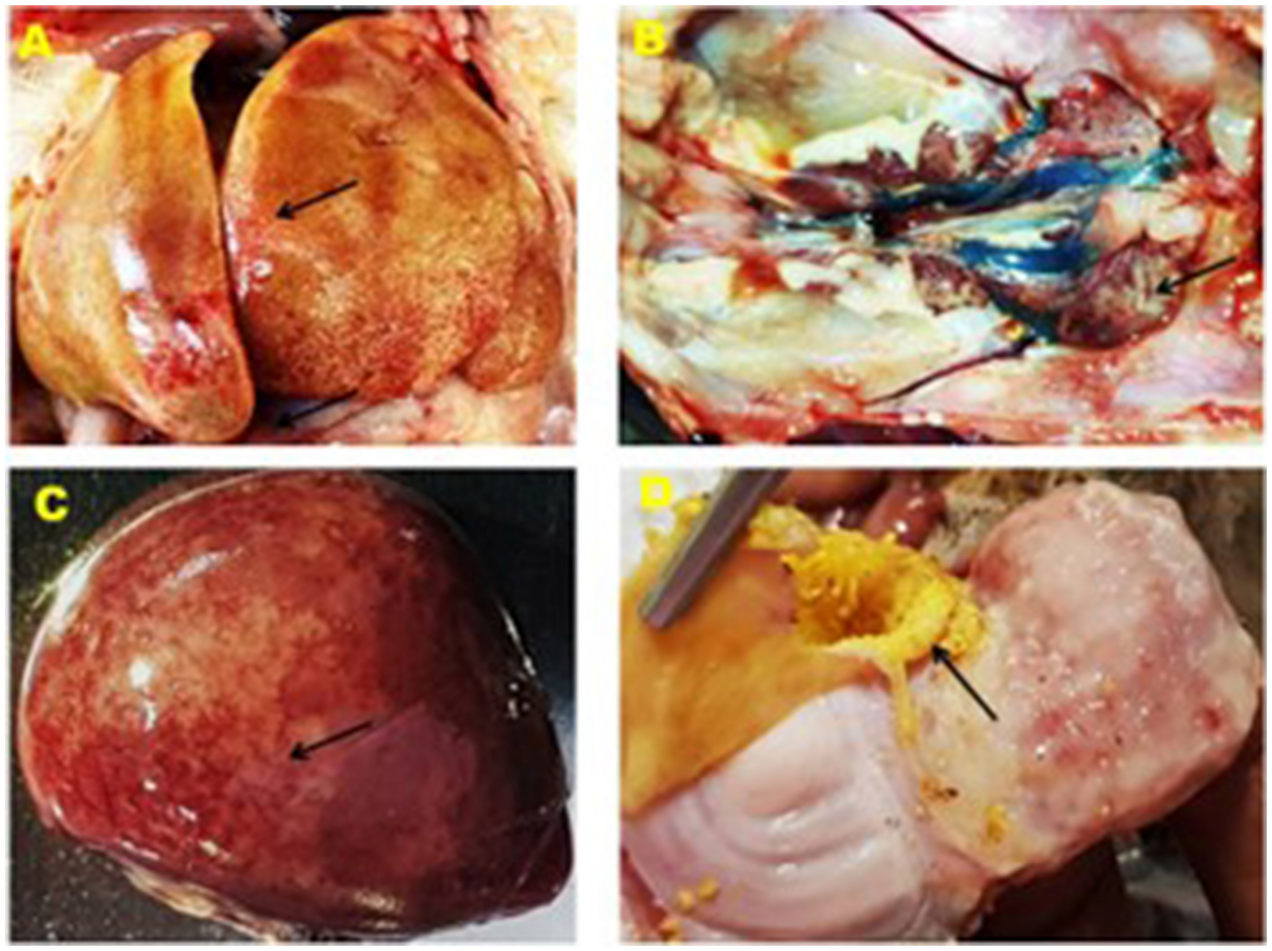
Gross lesion of the liver, kidney, spleen and gizzard of IBHV infected broiler chicken. **(A)** Black arrow showing white nodular and hemorrhagic spot in the liver, **(B)** black arrow showing swollen kidney, **(C)** black arrow showing white necrotic foci under the capsule of the spleen, and **(D)** black arrow showing erosion of the membrane in the gizzard.

Clinical signs observed in the chickens of affected farms were anorexia, depression, reluctance in movement, drowsiness, weight loss, ruffled feathers, lack of uniformity, respiratory distress, and death occurring 5–6 days after the onset of signs in birds of 3 to 6 weeks old. The postmortem investigation revealed that the liver of the affected chickens was enlarged and had a fragile and discolored appearance, ranging from yellow to tan. There were also patchy soft areas, as well as tiny hemorrhages under the liver's outer layer and within the tissue. Furthermore, there were instances of petechial hemorrhage and bruises beneath the outer layer and tissue of the liver, erosion of the inner layer of the gizzard, swollen and congested spleen, and swollen and hemorrhagic kidney (Figure 2).

### 3.2 Histopathological investigation of liver samples

**Figure 3:**
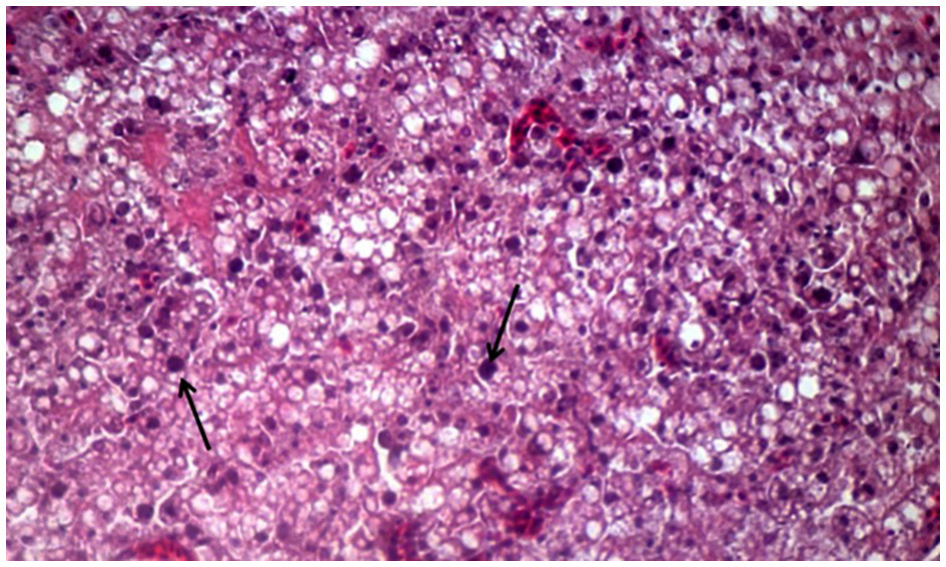
Histomorphology of the liver of IBHV-infected broiler chickens. Black arrow showing basophilic intranuclear inclusion bodies. H&E stain, bar indicates magnification.

Histopathological investigation of the liver samples of chickens infected naturally with suspected FAdV revealed the presence of severe and extensive hepatic necrosis, and basophilic intranuclear inclusion bodies in the hepatocytes (Figure 3).

### 3.3 Molecular detection the FAdV by PCR

**Figure 4:**
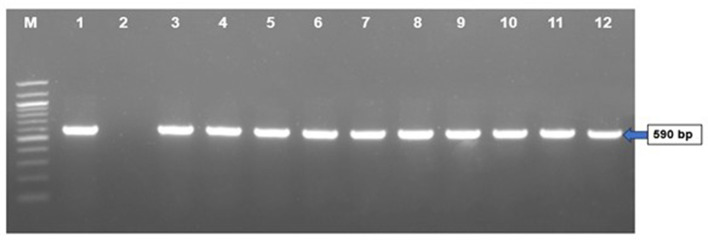
PCR amplification of hexon gene of FAdVs of field samples. Lane M = 100 bp DNA ladder, Lane 1 = positive control, Lane 2 = negative control, Lanes 3–12 = FAdV positive field samples.

The results of molecular detection by PCR confirmed that only 40 (46.51%) out of 86 liver samples were positive for FAdV (Table 2). The PCR performed using hexon gene primers showed a very clear band at the expected size of 590 bp in 1.5% agarose gel electrophoresis (Figure 4). These molecular assays have provided conclusive evidence that FAdV is the causative agent of IBH in broiler and layer bird samples collected during this study in Bangladesh.

**Table 2:** Molecular detection, sequence analysis (hexon genes) and isolation of FAdV from liver samples of broiler and layer chickens.

**Name of districts**	**No. of liver samples collected**		**PCR positive**		**Isolation positive using ECEs**		**Hexon gene sequencing of recent isolates**
	**Broiler**	**Layer**	**Broiler**	**Layer**	**Broiler**	**Layer**	
Feni	8	6	6	2	3	2	4
Tangail	7	5	4	1	2	0	1
Dhaka	10	5	5	2	2	1	2
Joypurhat	7	4	3	2	2	2	2
Gazipur	9	7	5	3	3	2	3
Mymensingh	6	4	2	2	2	0	-
Cumilla	5	3	2	1	1	0	-
**Total**	**52**	**34**	**27**	**13**	**15**	**7**	**12**

PCR, polymerase chain reaction; ECEs, Embryonated chicken eggs.

### 3.4 Isolation of FAdV using seronegative ECEs

**Figure 5:**
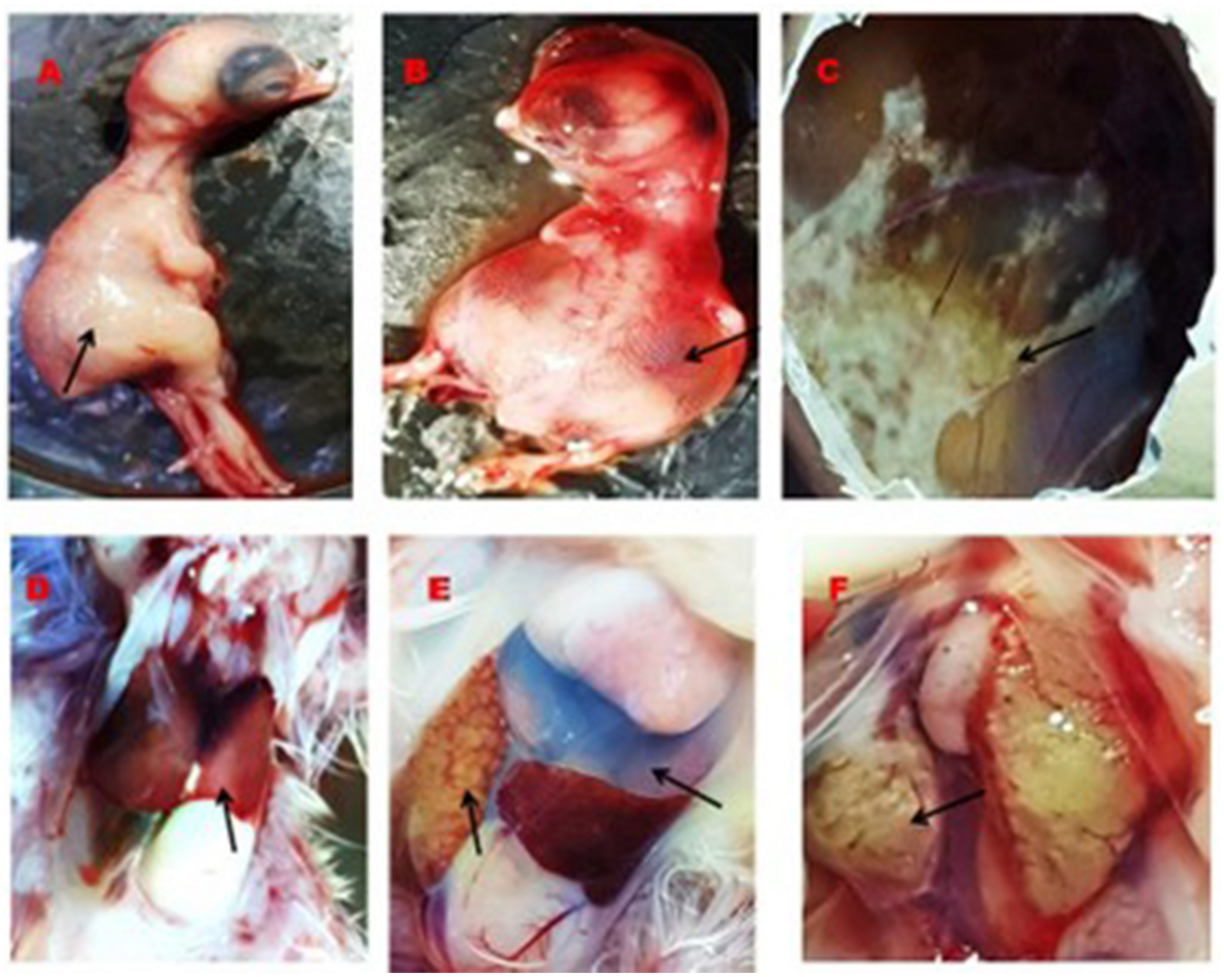
Gross lesion in the embryos, CAM, liver, heart, and kidney. **(A)** Normal embryo (black arrow), **(B)** accumulation of edematous fluid in the IBHV infected embryos (black arrow), **(C)** development of pock lesion on the CAM of IBHV infected embryos (black arrow), **(D)** normal structure of the liver of control embryos (black arrow), **(E)** white nodular lesion on the liver of virus infected embryos (black arrow) and accumulation of excess fluid in the pericardium of the virus infected embryos (upper black arrow), and **(F)** development of white nodular mass in the kidney of virus infected embryos (black arrow).

**Figure 6:**
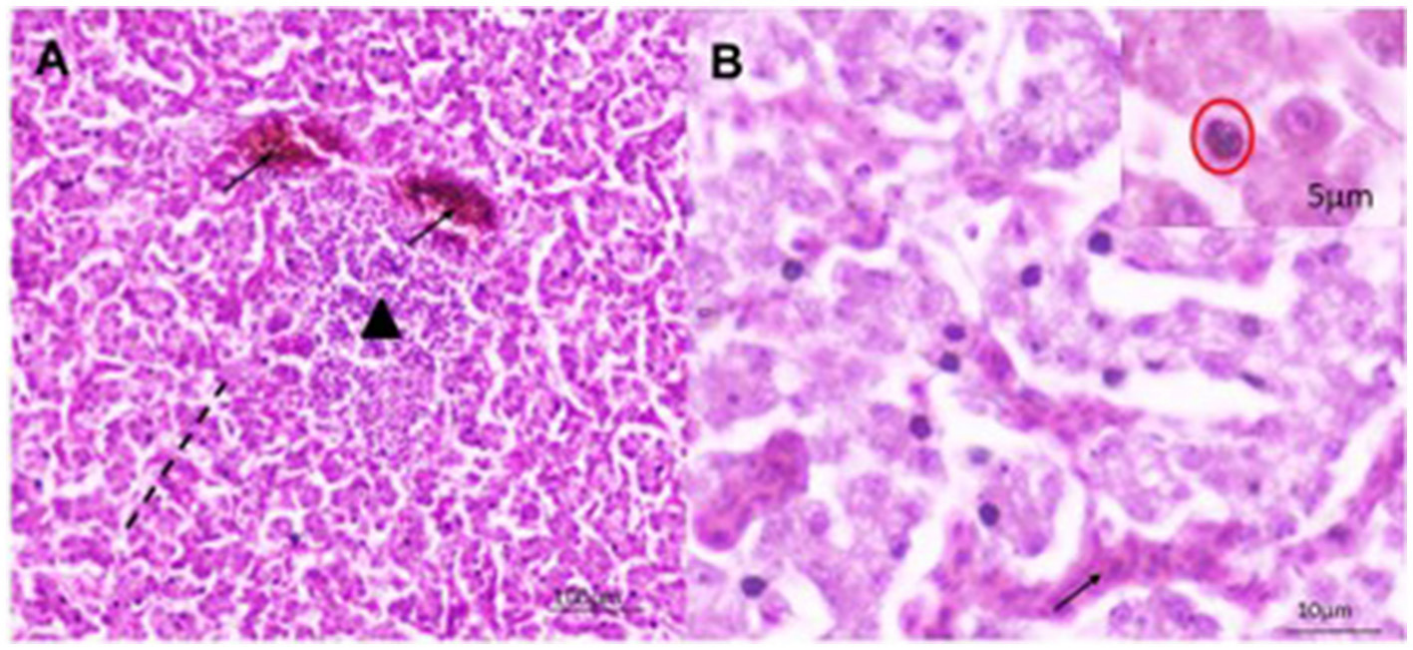
Histomorphology of the liver of IBHV-infected embryo. **(A)** Hepatocellular necrosis (area of black dash) with focal aggregation of mononuclear cells (black triangle) and congested blood vessels (black arrow); **(B)** several basophilic intranuclear inclusion bodies (red circle in inset box) in hepatocytes with discreet hemorrhages (black arrow). H&E stain, bar indicates magnification.

**Figure 7:**
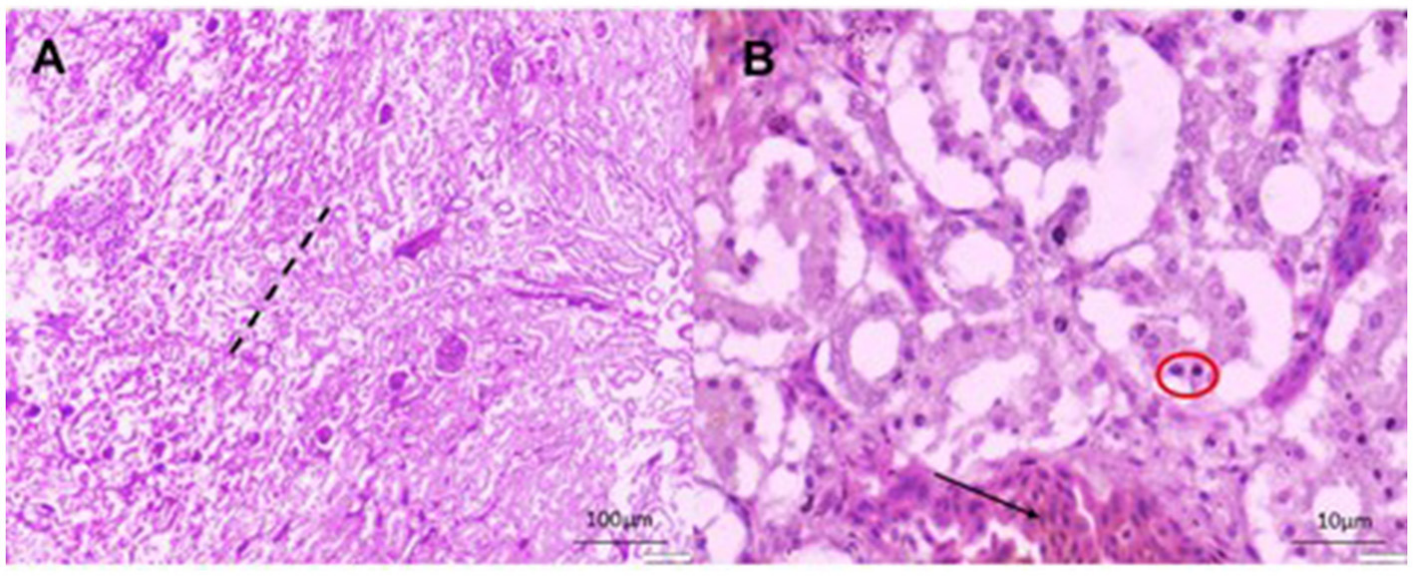
Histomorphology of the kidney of IBHV-infected embryo. **(A)** Degeneration and necrosis of tubular epithelium (area of black dash) with infiltration of mononuclear cells; **(B)** basophilic intranuclear inclusion bodies (red circle), focal hemorrhages (black arrow). H&E stain, bar indicates magnification.

A total of 22 (55%) out of 40 PCR-positive FAdV liver samples were found to be positive for IBHV isolation in seronegative ECEs (Table 2). The ECEs inoculated with PCR-confirmed samples killed the embryos within 3 to 4 days after post-inoculation. The viruses isolated from 22 samples were further confirmed by PCR using similar gene-specific primers. The embryos showed changes in the CAM by the development of pock lesions. Additionally, the embryos displayed hemorrhagic lesions and enlarged livers that exhibited either yellow to reddish foci or discoloration (Figure 5). Histological examination of the embryonic liver revealed acute hepatitis, which was identified by necrotic hepatocytes (Figure 6), and basophilic inclusion bodies along with tubular necrosis and congestion in the kidney tissue (Figure 7). Furthermore, there was excessive fluid accumulation in the pericardial sac of the embryonic heart, known as hydropericardium.

### 3.5 Amplification and sequencing of the hexon gene

The study successfully amplified the hexon gene of 12 recent isolates of FAdVs by PCR using gene-specific primers and sequenced 590 bp PCR products of those isolates. The nucleotide sequences of the hexon gene of the 12 isolates of FAdV were submitted to the GenBank database for accession numbers (PP897930, PP897931, PP897932, PP897933, PP897934, PP897935, PP858860, PP858861, PP858862, PP858863, PP817068, and PP817069).

### 3.6 Phylogenetic analysis

**Figure 8:**
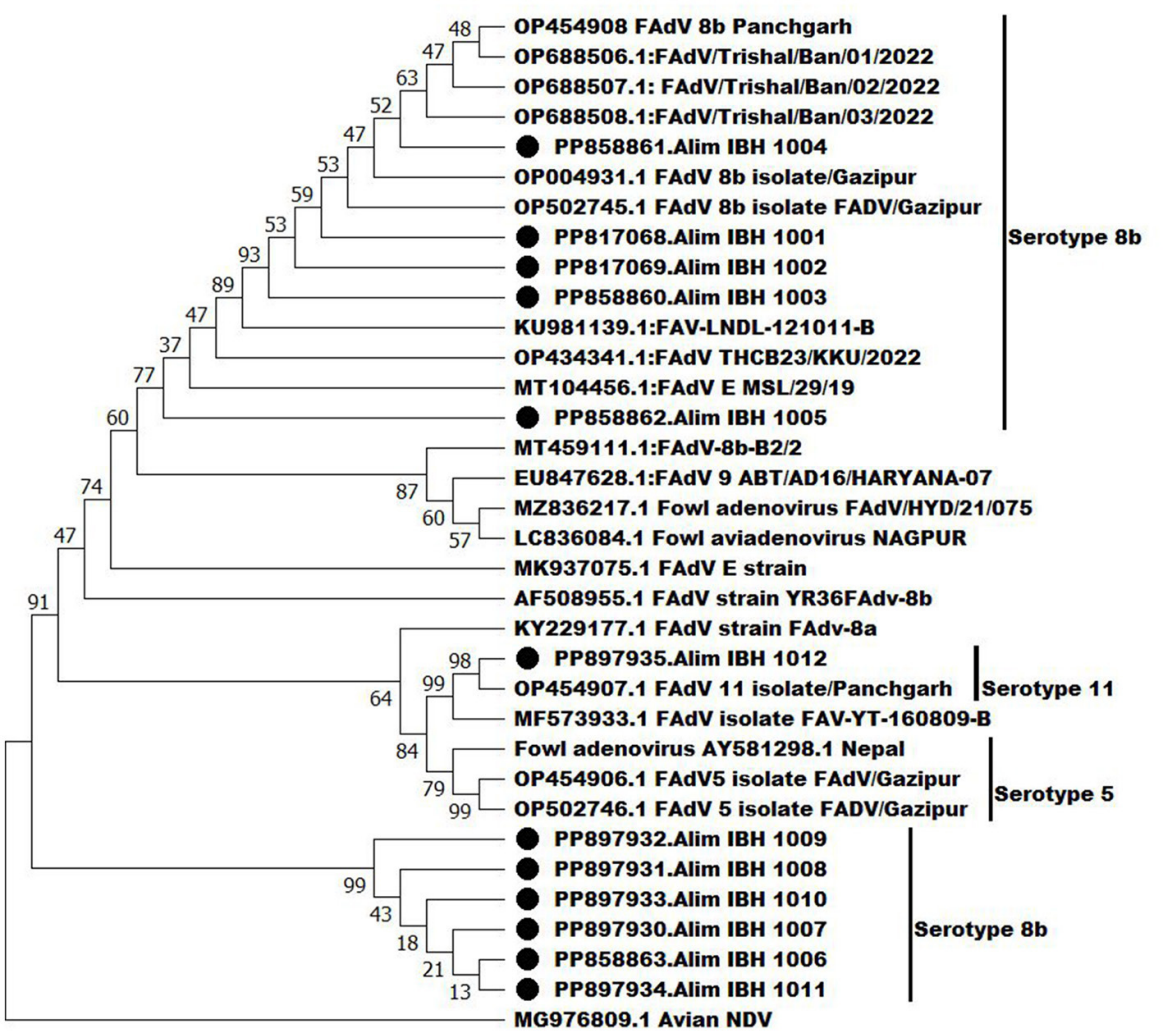
Phylogenetic analysis of the hexon genes of the recent isolates of FAdVs with the nucleotide sequences of the hexon genes of FAdV isolates retrieve from GenBank by neighbor-joining method and the evolutionary investigations were carried out using the MEGA 11 program.

Phylogenetic analysis using the hexon gene revealed that one FAdV isolate, Alim IBH 1012, belongs to the serotype 11 of FAdV-D (Figure 8). It shares 90% homology with a previous isolate of Bangladesh (OP454907.1FAdV11 isolate/Panchgarh). Among the recent isolates, six isolates (Alim IBH 1009, Alim IBH 1011, Alim IBH 1007, Alim IBH 1010, Alim IBH 1006, and Alim IBH 1008) clustered with serotype 8b of FAdV-E, showed a close relationship with the isolates of Turkey and India. On the other hand, three more recent isolates of the serotype 8b of FAdV-E (Alim IBH 1003, Alim IBH 1002, and Alim IBH 1001) formed a separate cluster with 8b isolates of Gazipur, Bangladesh (OP502745.1FAdV 8b isolate FADV/Gazipur). Similarly, the isolate Alim IBH 1005 showed close similarity to an isolate from Turkey (MK642682.1: FAdVM41), and the isolate Alim IBH 1004 from this study also showed close similarity with the previous isolate of Trishal, Bangladesh (OP688508.1: FAdV/Trishal/Ban/03/2022) (Figure 8). In phylogenetic analysis, the study observed that recent isolates of FAdV showed 95%−100% similarity with the previous isolates from Bangladesh and have more than 90% similarity with the isolates from India and Nepal. The study found that serotype 8b was isolated from both broiler and layer chickens, while serotype 11 was isolated exclusively from layer chickens. No differences were observed in the hexon gene analysis of the FAdVs isolated from broiler and layer chickens.

### 3.7 Pathogenicity assessment of the isolated FAdVs serotype 11 and 8b

**Figure 9:**
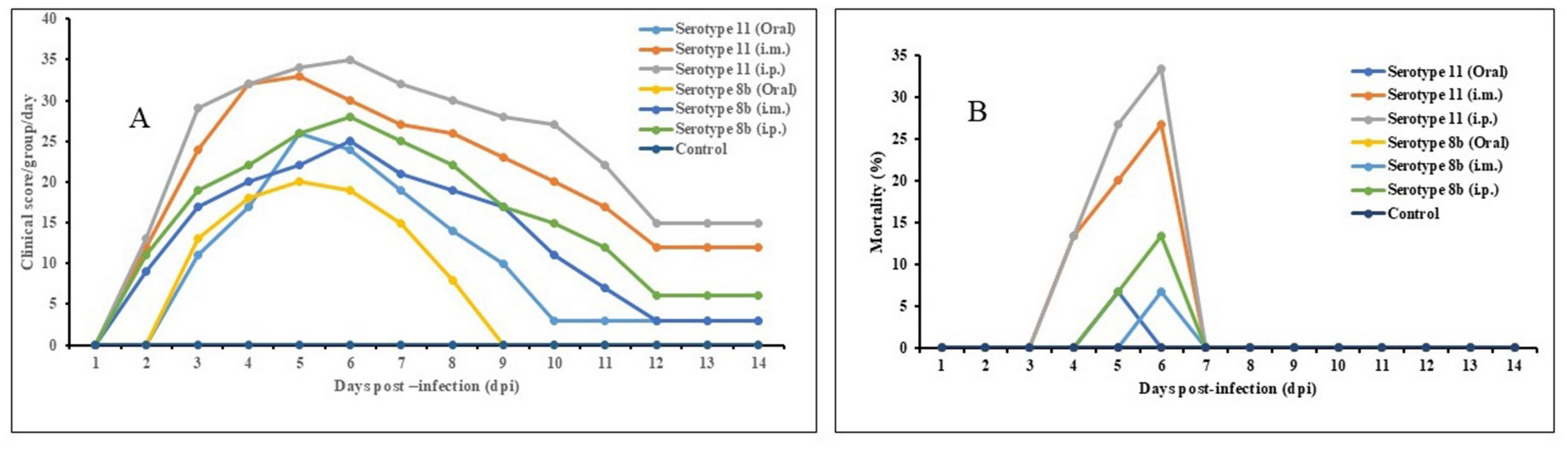
Pathogenicity of serotype 11 (Alim IBH 1012) and serotype 8b (Alim IBH 1010) strains in seronegative broiler chicks at different routes. **(A)** Clinical signs scores of seronegative chicks inoculated with serotype 11 (Alim IBH 1012) and serotype 8b (Alim IBH 1010) strains at different routes. **(B)** Day-wise mortality pattern of seronegative chicks inoculated with serotype 11 (Alim IBH 1012) and serotype 8b (Alim IBH 1010) strains at different routes.

Chickens infected with serotype 11 (Alim IBH 1012) and serotype 8b (Alim IBH 1010) through i.p., i.m., and oral routes exhibited typical clinical signs of FAdV/IBHV shown in Figure 9A. Chickens inoculated orally with FAdV exhibited a lower mortality rate compared to those infected via i.p. and i.m. routes. Clinical signs in orally inoculated chickens began at 3 days post-infection (dpi), peaked at 5 dpi, and gradually diminished by 8 dpi, with no apparent signs after 9 dpi. In contrast, chickens in the i.p. and i.m. groups showed clinical signs as early as 2 dpi, which peaked between 3 and 7 dpi, and steadily decreased until 11 dpi, with no noticeable clinical signs after 12 dpi.

**Figure 10:**
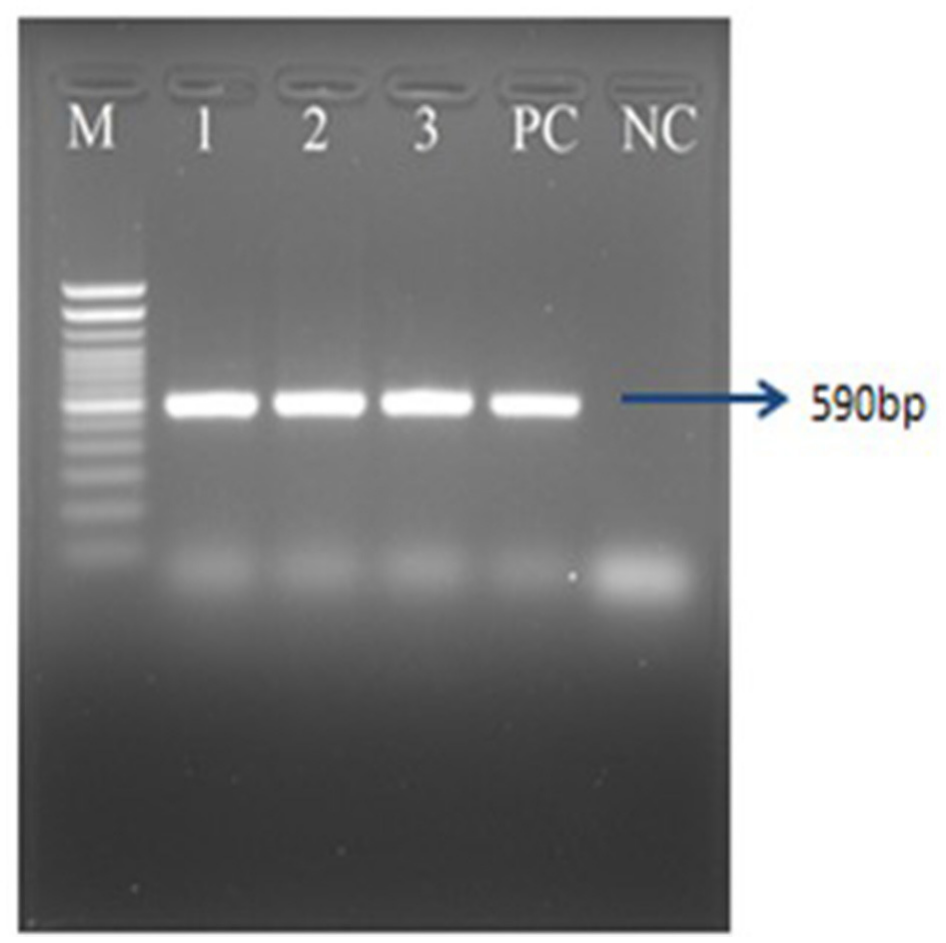
PCR amplification of hexon gene of FAdVs of the experimentally infected chicken. Lane M = 100 bp DNA ladder, Lane 1–3 = FAdV positive samples, PC, positive control; NC, negative control.

The chickens infected with serotype 11 (Alim IBH 1012), 6.67% died from oral virus inoculation, 26.67% died from i.m. inoculation, and 33.33% died from i.p. inoculation. For the chickens infected with serotype 8b (Alim IBH 1010), none of the chicken died (0%) from oral virus inoculation, 6.67% died from i.m. inoculation, and 13.33% died from i.p. inoculation (Figure 9B). The control group of chickens remained healthy throughout the investigation. Necropsy results showed enlarged, friable livers with pale to yellowish coloring, as well as the presence of necrotic foci. Chickens that died between experimental time points displayed similar pathological lesions as euthanized birds. No noticeable lesions were found in the tissues of mock-infected chickens. PCR analysis confirmed the re-isolation of FAdV from the experimentally infected broiler chickens (Figure 10).

## 4 Discussion

The FAdV or IBHV is causing significant disruptions in the poultry industry of Bangladesh, causing reduced growth rates, poor feed conversion ratio, and increased morbidity and mortality (Islam et al., 2023). This leads to production losses, higher costs to the farmers, and increased expenses for veterinary care. The disease also affects food security and price fluctuations, necessitating the implementation of preventive measures and effective management strategies for industry sustainability (Sohaimi and Clifford, 2021). For this reason, the present study was conducted for pathological investigation (gross and histopathology), molecular identification, and characterization of the circulating serotypes of different genotypes of FAdV (E and D) in the commercial poultry (broilers and layers) in Bangladesh.

In this study, samples were collected from FAdV-suspected commercial broiler and layer poultry based on clinical signs and postmortem examinations. Islam et al. (2023) conducted a similar study and reported various clinical signs such as depression, anorexia, drowsiness, reluctance to move, lack of uniformity, reduced body weight gain, ruffled feathers, respiratory distress, and high rate of morbidity and mortality (Cizmecigil et al., 2020). Postmortem examinations revealed hemorrhages in the skeletal muscles, an enlarged and friable liver with a yellow to tan color and mottled appearance, and localized soft areas (El-Shall et al., 2022). Additionally, petechial and ecchymotic hemorrhages were found beneath the capsule and in the parenchyma. Fluid accumulation in the pericardial sac, swollen and hemorrhagic kidneys, and enlarged and congested spleens and pancreas were also frequently observed in the dead birds (Chitradevi et al., 2021). The liver tissue of naturally infected chickens showed hepatocyte necrosis and basophilic intranuclear inclusion bodies (INIB), similar to the observations made by previous researchers (Oliver-Ferrando et al., 2017; Zhao et al., 2015). Additionally, the kidneys displayed signs of massive tubular necrosis, localized hemorrhages, and mononuclear cell infiltration (Mariappan et al., 2018; Chen et al., 2017).

The effective identification of FAdV was validated in this work utilizing PCR with gene specific primers that target the L1 loop of the hexon gene. This result aligns with the findings of prior research undertaken by Islam et al. (2023), Radwan et al. (2019), and El-Tholoth and Abou El-Azm (2019). FAdV was isolated from liver homogenates and CAM of embryonated chicken eggs. PCR confirmed FAdV in infected CAM, liver samples from dead commercial poultry, and inoculated embryos, aligning with previous studies (Hemida and Alhammadi, 2017). The presence of INIB in hepatocytes is a characteristic sign of FAdV in IBH infected chickens. INIB was detected in hepatocytes at 5–6 days of post-infection. This was accompanied by the existence of pock lesions, hydropericarditis, edema, an enlarged and hemorrhagic liver with yellow or reddish spots, and/or overall discoloration in the virus-infected embryos. Adenovirus-induced similar lesions were previously reported by Alemnesh et al. (2012).

The results of PCR and the isolation rate of FAdV from naturally suspected cases in this study revealed that FAdV typically exhibits lower pathogenicity in layer chickens than the broiler chickens, primarily due to differences in genetics, immune response, and management practices (Schachner et al., 2018). Layer chickens have more developed immune system, as they are bred for longer lifespans and gradual growth. This maturation enables them to mount a more effective defense against infections. In contrast, broiler chickens are selected for rapid growth and higher meat yield, which can weaken their immune capacity and make them more susceptible to diseases like FAdV (Brown Jordan et al., 2018). Additionally, the stress and crowded conditions commonly present in broiler production can increase their susceptibility to infections.

The phylogenetic analysis showed that six recent FAdV serotype 8b isolates formed a distinct cluster, similar to isolates from India and Turkey, while the remaining five serotype 8b isolates were similar to previous Bangladeshi isolates. Additionally, one isolate of serotype 11 (FAdV-E), showed close similarities with a previous isolate of Bangladesh that was also reported by Islam et al. (2023). Most of these outbreaks of FAdVs in the commercial poultry of Bangladesh have been associated with the genotypes FAdV-D or -E, also aligned with the serotypes 2, 11, 8a, and 8b (Hosseini et al., 2020). It is anticipated that various serotypes of FAdV have been introduced into the commercial poultry population of Bangladesh since the initial outbreak was reported in 2002. The increased similarity among serotypes 8b also indicates that their transmission in commercial poultry might be from inside the country. The poultry business in Bangladesh was founded by importing birds, eggs, feed ingredients, and utensils from countries where FAdVs had been reported as endemic. The importation of these materials may have enabled the introduction of FAdVs in Bangladesh. Moreover, it is worth noting that FAdVs can transmit vertically, meaning through the embryonated egg from one generation to the next (Li et al., 2017).

The pathogenicity study showed that serotype 11 caused slightly higher bird mortality than serotype 8b across intraperitoneal, intramuscular, and oral routes of infection. The findings also align with those of Islam et al. (2023), who also reported higher mortality rates of chickens infected with serotype 11 of FAdV compared to serotypes 8b and 5. Additionally, birds infected simultaneously with FAdV serotypes 8b and 11 showed more severe clinical signs and higher mortality rates than those inoculated with serotypes 2, 7, and 8a (Matos et al., 2016). The mortality rate of orally-infected chickens with FAdV in this study was lower compared to the chickens infected via the i.m. and i.p. routes. The mortality rates observed through the i.m. route closely related to the findings of a study conducted by Wang et al. (2023). According to their research, chickens that were experimentally infected with serotype 8b had a mortality rate of 10%, while those infected with serotype 11 had a mortality rate of 30%. However, the mortality rate for chickens infected with serotype 11 through the oral route was only 5% according to Abghour et al. (2021). In general, i.p. inoculation leads to a faster and more extensive infection. This is because the virus is directly introduced into the body cavity, allowing it to quickly reach internal organs and the circulatory system (Al Shoyaib et al., 2020). As a result, clinical signs appear more rapidly, and the infection rate is higher compared to i.m. injection.

## 5 Conclusions

The study found that FAdV infection is common in commercial broiler and layer chickens of Bangladesh and is responsible for their substantial rate of morbidity and mortality. The identification of FAdVs using PCR and isolation of the viruses using ECEs was done successfully. The phylogenetic analysis of the study indicated that commercial broilers and layer birds are infected with FAdVs serotypes 11 and 8b, which belong to genotypes D and E. Serotype 8b was shown to be the predominant serotype of the two sero-types of the FAdV. Between the two serotypes, serotype 11 was found to be more pathogenic than serotype 8b based on clinical signs and rate of morbidity and mortality during experimental infection. The study also indicated that the mortality rate was found to be higher in the i.p. and i.m. routes compared to the oral route in experimental infection. Further research is needed to understand the details of variation in pathogenicity of these circulating FAdVs and also to develop live and killed FAdV vaccines using locally circulating serotypes of FAdV isolates of Bangladesh.

## Data Availability

The datasets presented in this study can be found in online repositories. The names of the repository/repositories and accession number(s) can be found in the article/Supplementary material.
